# Dry Reforming of Methane Using Gd-promoted Ni/SBA-16 Catalyst: Structure, Activity and Process Optimization with Response Surface Methodology

**DOI:** 10.3390/nano15191527

**Published:** 2025-10-06

**Authors:** Salma A. Al-Zahrani, Mohammed F. Alotibi, Ahmed I. Osman, Ahmed A. Bhran, Maha Awjan Alreshidi, Ahmed Al Otaibi, Hessah Difallah A. Al-Enazy, Nuha Othman S. Alsaif, Ahmed S. Al-Fatesh

**Affiliations:** 1 Chemistry Department, Faculty of Science, University of Ha’il, P.O. Box 2440, Ha’il 81451, Saudi Arabia; m.alrashedi@uoh.edu.sa (M.A.A.); ahmed.alotaibi@uoh.edu.sa (A.A.O.); h.alenazy@uoh.edu.sa (H.D.A.A.-E.); n.alseif@uoh.edu.sa (N.O.S.A.); 2 Scientific and Engineering Research Center, University of Ha’il, Ha’il 2440, Saudi Arabia; 3 Institute of Refining and Petrochemicals Technologies, King Abdulaziz City for Science and Technology (KACST), P.O. Box 6086, Riyadh 11442, Saudi Arabia; 4 School of Sciences, Psychology, Arts and Humanities, Computing, Engineering & Sports, Canterbury Christ Church University, Canterbury CT1 1QU, UK; ahmed.osman@canterbury.ac.uk; 5 Chemical Engineering Department, College of Engineering, Imam Mohammad Ibn Saud Islamic University (IMSIU), Riyadh 11432, Saudi Arabia; aabahran@imamu.edu.sa; 6 Chemical Engineering Department, College of Engineering, King Saud University, P.O. Box 800, Riyadh 11421, Saudi Arabia

**Keywords:** Ni/SBA-16 catalyst, gadolinium promoter, dry reforming of methane, response surface methodology (RSM), catalyst optimization, hydrogen production

## Abstract

This work examines the effect of gadolinium (Gd) promotion on nickel-based SBA-16 catalysts for the dry reforming of methane (DRM), with the goal of improving syngas production by optimizing catalyst composition and operating conditions. Catalysts with varying Gd loadings (0.5–3 wt.%) were synthesised using co-impregnation. XRD, N_2_ physisorption, FTIR, XPS, and H_2_-TPR–CO_2_-TPD–H_2_-TPR were used to examine the structural features, textural properties, surface composition, and redox behaviour of the catalysts. XPS indicated formation of enhanced metal–support interactions, while initial and post-treatment H_2_–TPR analyses showed that moderate Gd loadings (1–2 wt.%) maintained a balanced distribution of reducible Ni species. The catalysts were tested for DRM performance at 800 °C and a gas hourly space velocity (GHSV) of 42,000 mL g^−1^ h^−1^. 1–2 wt.% Gd-promoted catalysts achieved the highest H_2_ (~67%) and CO yield (~76%). Response surface methodology (RSM) was used to identify optimal reaction conditions for maximum H_2_ yield. RSM predicted 848.9 °C temperature, 31,283 mL g^−1^ h^−1^ GHSV, and a CH_4_/CO_2_ ratio of 0.61 as optimal, predicting a H_2_ yield of 96.64%, which closely matched the experimental value of H_2_ yield (96.66%). The 5Ni–2Gd/SBA-16 catalyst exhibited minimal coke deposition, primarily of a graphitic character, as evidenced by TGA–DSC and Raman analyses. These results demonstrate the synergy between catalyst design and process optimization in maximizing DRM efficiency.

## 1. Introduction

Hydrogen (H_2_) is widely recognised as a cornerstone of the clean energy transition, due to its zero carbon emissions and versatility across both industrial and energy sectors. Current global H_2_ demand reached more than 97 million tonnes per year in 2023 [1]. The major processes for H_2_ production include steam methane reforming, electrolysis, and biomass gasification [2]. H_2_ must be produced through sustainable pathways to make a significant contribution to the global energy transition [3]. Dry reforming of methane (DRM) is an effective process that reduces two major greenhouse gases, i.e., carbon dioxide (CO_2_) and methane (CH_4_), while producing a mixture of carbon monoxide (CO) and H_2_, a valuable syngas stream [4,5]. The DRM reaction is represented as:

(1) $$CH_{4}+CO_{2}\to 2CO+2H_{2}\;\Delta H_{298k}^{o}=247.3\;kJ\cdot moL^{-1}$$

One of the key benefits of DRM is that it produces syngas with an H_2_:CO ratio close to 1:1, which supports the stoichiometric requirements of processes including Fischer–Tropsch synthesis and methanol production, and can be further processed to obtain high-purity H_2_ [6]. DRM is a highly endothermic reaction and typically needs high temperatures (700–900 °C) to achieve significant conversions [7]. Under such conditions, the conventional catalysts are prone to deactivation via sintering of metal particles and deposition of carbonaceous species, resulting in a gradual decline in activity [8].

Ni-based catalysts are commonly used in DRM due to their relatively low cost, high intrinsic activity for CH_4_ and CO_2_ activation, and industrial scalability. However, their performance can be significantly enhanced using suitable supports and promoters [9]. An ideal support should have a high surface area for Ni dispersion, thermal stability under DRM conditions, and regulate the redox properties of the catalyst system [10]. Silica-based supports have gained attention due to their surface area, ease of surface modification, and inert nature. SBA-16 is a 3D mesoporous silica material characterized by its cage-like pores. It has emerged as a promising support for Ni-based catalysts in recent years. Its structure offers good thermal stability and allows uniform dispersion of active metal nanoparticles, which are essential for DRM [11,12].

Promoters are added to catalysts to enhance their stability, activity, and resistance to deactivation. The structure of SBA-16 is reported to synergize effectively with redox promoters, particularly with rare-earth metals, to enhance catalytic performance and stability. In the study by Zhang et al. (2013), the cerium-promoted Ni/SBA-16 achieved high conversions and catalyst stability over 100 h [13]. Huo et al. (2017) modified SBA-16 via ammonia nitridation and reported that N incorporation improved Lewis basicity and metal–support interaction and resulted in higher activity compared to unpromoted Ni/SBA-16 [14]. Taherian et al. (2021) studied Ni/SBA-16 systems promoted with yttrium, cerium, and lanthanum over MgO-modified SBA-16 and observed enhanced CO_2_ activation, improved metal dispersion, and reduced coke formation [11]. Among the rare-earth elements, Gadolinium (Gd) is known for its high capacity for oxygen storage, strong redox behaviour, and its capability to form thermally stable oxides [15]. The promotional effects of Gd are studied across various catalyst supports. The Gd loadings on Ni/MCM-41 improved CH_4_ and CO_2_ conversions up to ~87% and ~91%, respectively, even at 0.1 wt.% loading. Catalysts maintained a H_2_/CO ratio of ~1 by enhancing Ni dispersion and generating surface oxygen vacancies [16]. Similarly, Zhang et al. showed that promotion of Gd to mesoporous Ni/Al_2_O_3_-CeO_2_ catalysts enhanced the catalytic stability over prolonged operation [17]. Fakeeha et al. (2023) in their studies demonstrated that Gd-promoted Ni/YZr catalysts achieved high H_2_ yield [15,18]. These studies emphasized that the Gd promoter can effectively improve the activity of Ni-based catalysts with various supports.

In addition to proper catalyst design, optimizing operating conditions is essential to bridge the gap between experimental DRM performance and practical, industrial applications. Response Surface Methodology (RSM) is a statistical method that allows systematic evaluation of multiple operating variables and their interactions, enabling the identification of optimum conditions with fewer experiments [19]. RSM is generally applied to balance operating feed ratio, temperature, and gas hourly space velocity (GHSV) to optimize performance parameters in DRM [20].

Although Ni catalysts, SBA-16 supports, and rare-earth promoters have been investigated individually, the combined effect of Gd incorporation into Ni/SBA-16 systems remains unexplored. The present work addresses this gap by systematically examining how Gd influences the structural and redox properties of Ni/SBA-16 and correlating these changes with catalytic performance in DRM. Gd-promoted Ni/SBA-16 catalysts with varying Gd loadings (0.5–3 wt.%) were synthesized via co-impregnation. Catalytic activity was evaluated in terms of H_2_, CO yields, and H_2_/CO ratio under DRM conditions at 800 °C and a GHSV of 42,000 mLg^−1^ h^−1^. The novelty of this work lies in integrating Gd-promoted Ni/SBA-16 catalysts with RSM-based process optimization for DRM. This approach may provide a statistical understanding of how reaction parameters influence catalyst properties and offer a predictive framework for identifying operating conditions that maximize H_2_ and CO yields.

## 2. Materials and Methods

### 2.1. Materials

Ni (NO_3_)_2_.6H_2_O (Nickel nitrate; 98%, Alfa Aesar); Gd (NO_3_)_3_.6H_2_O (Gadolinium nitrate; 98%, Alfa Aesar); SBA-16 (99.9%, Sigma-Aldrich, Darmstadt, Germany), and distilled water are used to prepare catalysts.

### 2.2. Catalyst Synthesis

A one-step wet impregnation process was used to prepare the Ni+xGd/SBA-16 catalysts. First, the solution was prepared by dissolving different amounts of Gd (NO_3_)_3_ 6H_2_O (to obtain 0, 0.5, 1, 2, and 3 wt.% Gd) and a calculated amount of Ni (NO_3_)_2_ 6H_2_O for a final loading of 5 wt.%. Ni in 30 mL of water. The SBA-16 support was subsequently impregnated with this mixture. Stirring was maintained until the liquid evaporated and the mixture thickened into a paste, ensuring uniform distribution of metal precursors. The prepared samples were dried at 120 °C for 12 h, followed by calcination in air at 600 °C for 3 h (heating rate 10 °C min^−1^) to obtain the final catalysts. The catalysts were designated as 5Ni+xGd/SBA-16, where x indicates the wt.% of Gd.

### 2.3. Catalyst Characterization Description

Catalyst characterization details are given in the Supplementary Material (S1).

### 2.4. Catalyst Evaluation

Catalytic testing for DRM was conducted in a stainless steel fixed-bed tubular reactor (PID Eng & Tech micro-activity reactor, Madrid, Spain) with an internal diameter of 0.94 cm and a length of 30 cm. A 0.1 g portion of undiluted catalyst powder was positioned over a glass wool plug inside the reactor, with a thermocouple placed in direct contact with the catalyst bed to monitor temperature. Before the reaction, the catalyst was reduced in a H_2_ flow (30 mL min^−1^) at 700 °C for 1 h. The reactor was then purged with nitrogen (20 mL min^−1^) to remove residual H_2_, and GC measurements confirmed its absence while heating to 800 °C under nitrogen flow. The DRM reaction was carried out at 1 bar with a CH_4_/CO_2_/N_2_ feed ratio of 3:3:1 (corresponding to 30, 30, and 10 mL min^−1^, respectively), giving a total flow of 70 mL min^−1^ and a GHSV of 42,000 mLg^−1^ h^−1^. Product analysis was performed using an online gas chromatograph (Shimadzu GC-2014, Shimadzu Corporation, Tokyo, Japan) equipped with molecular sieve and Porapak Q columns in combination with a thermal conductivity detector (TCD). The H_2_ yield, CO yield, and H_2_/CO were calculated using following equations:

(2) $$H_{2}Yield(\%)=\left(\frac{mol\;of\;H_{2}\;produced}{2\times mol\;of\;CH_{4}\;fed}\right)\times 100$$

(3) $$CO\;Yield(\%)=\left(\frac{mol\;of\;CO\;produced}{mol\;of\;CH_{4}\;fed+mol\;of\;CO_{2}\;fed}\right)\times 100$$

(4) $$\frac{H_{2}}{CO}ratio=\frac{mol\;of\;H_{2}\;produced}{mol\;of\;CO\;produced}$$

### 2.5. Experimental Design and Modelling Approach

#### 2.5.1. Central Composite Design (CCD)

Central Composite Design (CCD) is a widely used statistical design under the RSM framework that combines factorial runs, axial (star) points, and center points to model both linear and quadratic effects. This design enables efficient estimation of curvature, interactions among process variables, and provides rotatability, which ensures uniform prediction variance across the experimental space [21,22]. Due to these advantages, CCD has been extensively applied in the optimization of catalytic processes where nonlinear effects are expected. CCD was used to study the individual and interactive effects of GHSV, reaction temperature, and the molar ratio of CH_4_/CO_2_. The design included three types of runs. First, eight factorial points (2^3^ full factorial) were set at the low (−1) and high (+1) coded levels of each factor (Table 1).

Six axial points were added along each factor’s axis at a distance (α) of approximately 1.682 from the centre to capture the non-linear, quadratic effects required for a second-order model, thus ensuring rotatability and uniform prediction variance. Three replicate centre points were included, with all factors set at their midpoint (coded level 0), to evaluate experimental error and identify curvature in the response surface. The factor ranges were standardized by taking the midpoint of each factor as the central value, with deviations used to define variation limits, thereby improving the interpretability of the regression. The complete experimental design matrix is presented in Table 2.

#### 2.5.2. Quadratic Polynomial Regression Model

A second-order polynomial regression model was employed to describe the relationship between the input variables and the response. Higher-order models (e.g., cubic) were not chosen to avoid overfitting and unnecessary complexity. The general form of the quadratic regression model is expressed as:

(5) $$\hat{Y}=\beta_{0}+\sum_{i=1}^{3}\beta_{i}X_{i}+\sum_{i=1}^{3}\beta_{ii}X_{i}^{2}+\sum_{i=1}^{2}\sum_{j=i+1}^{3}\beta_{ij}X_{i}X_{j}+\varepsilon$$

here $\hat{Y}$ is the predicted response, X_i_ and X_j_ are the coded input variables, β_0_ is the intercept, β_i_ are the linear coefficients, β_ii_ are the quadratic coefficients (capturing curvature), β_ij_ are the interaction coefficients (accounting for combined effects), and ε is the random error term.

#### 2.5.3. Model Validation and Analysis

The adequacy of the model was assessed using analysis of variance (ANOVA). Model quality was assessed through R^2^, adjusted R^2^, and *p*-values, with terms having *p* < 0.05 considered statistically significant at the 95% confidence level. Residual analysis was conducted to verify assumptions of normality, independence, and constant variance. Predictive accuracy was confirmed by comparing experimental and predicted values, with correlations illustrated in parity plots.

## 3. Results and Discussion

### 3.1. Catalyst Characterization Results

#### 3.1.1. X-Ray Diffraction (XRD) Analysis

**Figure 1:**
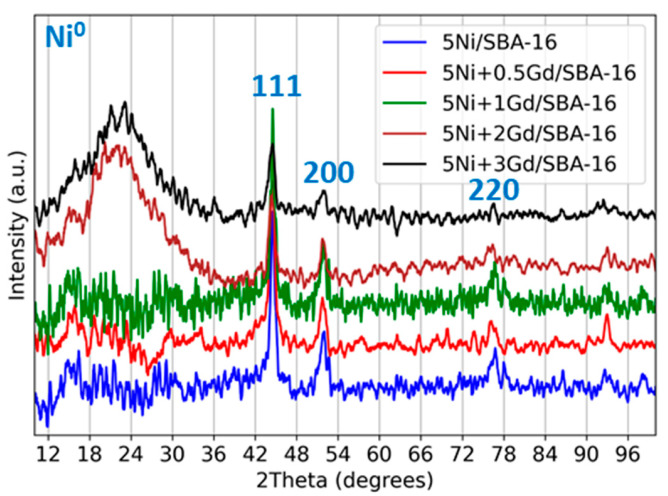
XRD patterns of reduced 5Ni+xGd/SBA-16 catalysts (x = 0, 0.5, 1, 2, 3 wt.%).

XRD patterns of the reduced 5Ni+xGd/SBA-16 (x = 0, 0.5, 1, 2, 3 wt.%) are presented in Figure 1.

It is observed from Figure 1 that all catalysts exhibited a diffuse hump in the 2θ range of 15–30° corresponding to amorphous silica from the support SBA-16 [23]. All catalyst samples exhibited three distinct diffraction peaks at 2θ ≈ 44.5°, 51.8°, and 76.3°, corresponding to the (111), (200), and (220) planes, respectively, for metallic Ni^0^ (face-centred cubic) (reference PDF No. 00-004-0850). In the unpromoted 5Ni/SBA-16 sample, the Ni^0^ peaks are comparatively sharp and intense. However, a slight decrease in peak intensity can be observed upon Gd addition, indicating the formation of smaller Ni crystallites and their improved dispersion [24]. No distinct diffraction peaks corresponding to Gd were observed in the 2θ range of (e.g., ~29°, 33°, 47.5°, 56°), suggesting Gd species are highly dispersed, amorphous, or below the detection limits.

#### 3.1.2. N_2_ Adsorption–Desorption Isotherms

**Figure 2:**
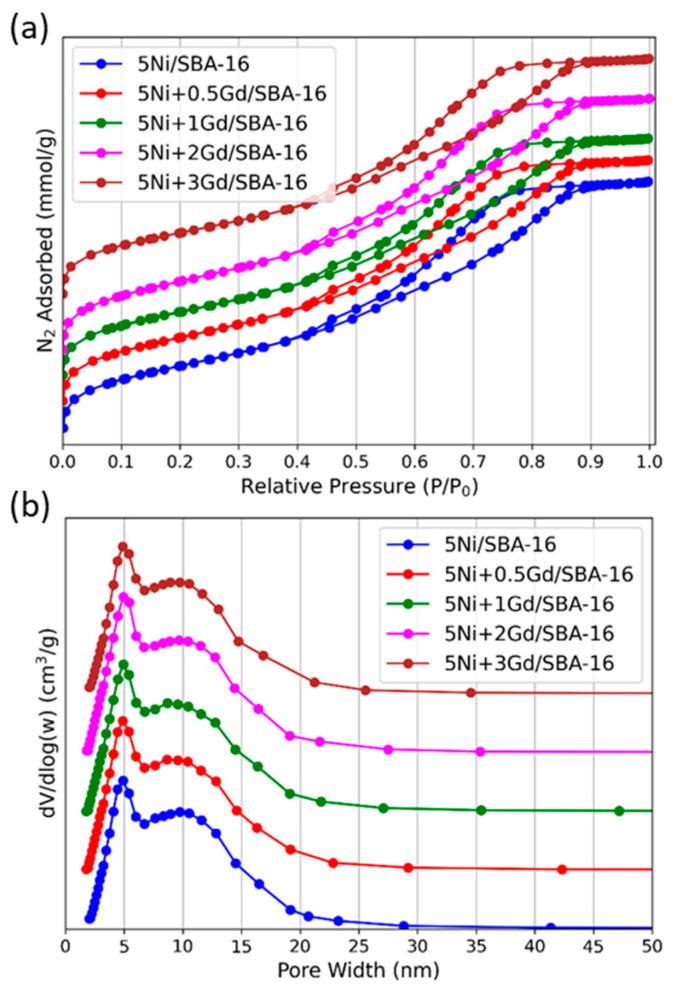
(**a**) N_2_ adsorption–desorption isotherms and (**b**) corresponding pore size distribution curves of the reduced 5Ni+xGd/SBA-16 (x = 0, 0.5, 1, 2, 3 wt.%).

The N_2_ adsorption–desorption isotherms of 5Ni+xGd/SBA-16 (x = 0, 0.5, 1, 2, 3 wt.%) are depicted in Figure 2a. The associated pore size distribution curves are presented in Figure 2b. All 5Ni+xGd/SBA-16 catalysts exhibited type IV isotherms with H2(b) hysteresis loop (H2(b) being the IUPAC classification for loops typically associated with cage-like mesoporous structures or ‘ink-bottle’ pores). Figure 2a characteristic of mesoporous materials with cage–like pores and interconnected networks, as in SBA-16. The corresponding BJH pore size distribution curves (Figure 2b) show narrow, symmetric peaks centred in the mesopore range (5–6 nm), with no significant broadening or shift upon Gd addition. This indicated the preservation of the ordered mesoporous structure of SBA-16 after Ni and Gd incorporation [25].

#### 3.1.3. FTIR Analysis

The FTIR spectrum of fresh 5Ni+xGd/SBA-16 catalysts (Figure S1, Supplementary Information) showed typical characteristic vibrations of the SiO_2_ framework and surface hydroxyl groups. The strong band observed around 1080–1250 cm^−1^ corresponds to the asymmetric stretching of Si–O–Si bonds. A medium-intensity band near ~800 cm^−1^ is assigned to the symmetric stretching of Si–O–Si, while the weak band around 460–470 cm^−1^ corresponds to Si–O–Si bending modes [26].

Surface hydroxyl groups are indicated by a sharp band at ~3745 cm^−1^, attributed to isolated silanol (Si–OH) groups, and a broad band between 3200–3650 cm^−1^ due to hydrogen-bonded –OH groups or adsorbed water. Additionally, the -OH bending mode appeared as a weak band near 1630 cm^–1^. The presence of silanol groups on the surface suggested potential sites for functionalization or metal anchoring, while the characteristic Si–O–Si bands confirmed that the mesoporous silica framework is preserved after calcination during catalyst synthesis. No distinct peaks of NiO or Gd_2_O_3_ were observed due to overlapping and masking from SiO_2_ peaks [27].

#### 3.1.4. X-Ray Photoelectron Spectroscopy (XPS) Analysis

**Figure 3:**
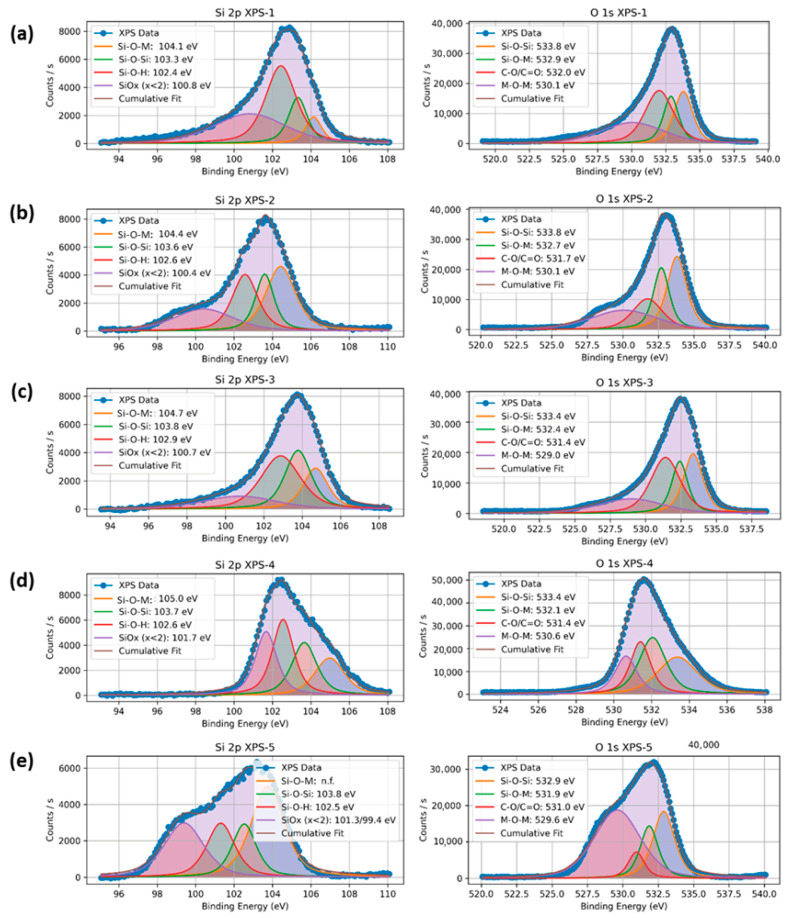
XPS spectra of the catalysts concerning Si 2p, and O 1s, (**a**) 5Ni/SBA-16, (**b**) 5Ni+0.5 Gd/SBA-16, (**c**) 5Ni+1Gd/SBA-16, (**d**) 5Ni+2Gd/SBA-16 and (**e**) 5Ni+3Gd/SBA-16.

XPS analysis was carried out to examine the surface chemical states, metal–support interactions, and electronic environment of Ni and Gd in the SBA-16-supported catalysts. Deconvoluted spectra of Si 2p and O 1s are shown in Figure 3, and the corresponding peak positions and area contributions are listed in Table 3a,b.

The Si 2p spectra (Figure 3) were fitted into four components: (i) Si–O–M (metal–support interaction) at ~104.1–105.0 eV, (ii) Si–O–Si (siloxane) at ~103.3–103.8 eV, (iii) Si–O–H (silanol) at ~102.4–102.6 eV, and (iv) SiOx (x < 2) at ~100.4–101.7 eV. Similar assignments have been reported in earlier XPS studies on mesoporous silica-based systems [28], [23]. For 5Ni/SBA-16, the Si–O–M (M = Ni or Gd) fraction was 8.1%. With 0.5 wt.% Gd loading, this increased to 33.4% and remained relatively high at 1 and 2 wt.% Gd loading (18.7–19.6%). This indicated the formation of Si-O-(Ni/Gd) linkages.

At 3 wt.% Gd, the Si-O-M component was absent, while Si–O–Si increased to 37.9% and Si–O–H decreased from 42.1% to 16.8%, suggesting surface dehydroxylation and a reduced density of interfacial bonds. The SiO_x_ contribution showed no consistent trend. This may be attributed to local disorder in the amorphous silica network [29].

The O 1s spectra contained Si–O–Si (~533.8–532.9 eV), Si–O–M (~532.9–531.9 eV), C–O/C=O (~532.0–531.0 eV), and a lattice-oxygen band at ~529–530.6 eV. The latter is assigned to overlapping Ni–O and Gd–O contributions, as the binding energies for NiO and Gd_2_O_3_ lattice-oxygen are similar and cannot be distinguished from O 1s alone. At 3 wt.% Gd, the lattice-oxygen fraction increased to 50.1%, indicating higher surface oxide coverage [9,29,30].

This trend is in line with the observed decrease in the C–O/C=O contribution (33.8% to 7.6%) and the reduction in Si–O–H in Si 2p at higher Gd loadings. Variations in Si–O–Si and Si–O–M contributions did not follow a clear trend; however, it suggested that the excessive Gd promoted oxide coverage or aggregation, thereby reducing the number of effective metal–support linkages [28,31].

#### 3.1.5. H_2_-TPR and Post-CO_2_-TPD Analysis

**Figure 4:**
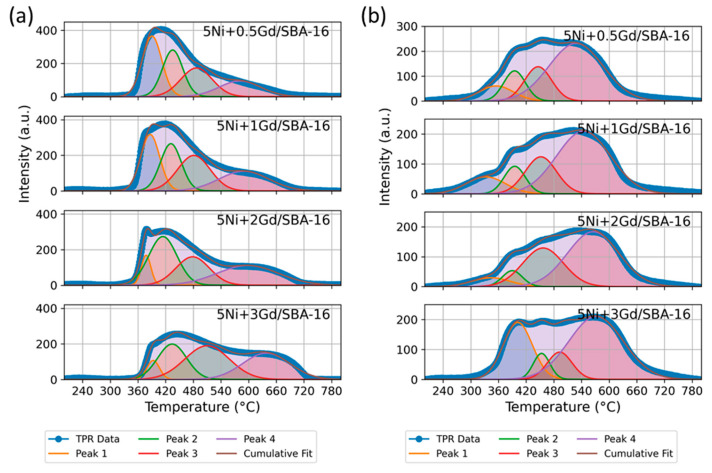
(**a**) H_2_–TPR reduction profiles of fresh and (**b**) post–CO_2_–TPD H_2_–TPR profiles for 5Ni +xGd/SBA-16 catalysts (x = 0.5, 1, 2, 3 wt.%).

H_2_–TPR was performed to investigate the reduction behaviour of Gd-promoted Ni/SBA-16 catalysts. The reduction profiles for the fresh catalysts are presented in Figure 4a, and the corresponding peak temperatures and relative areas are summarized in Table 4.

Four main reduction peaks were identified. Peak 1 (~377–393 °C) was attributed to the reduction of free or weakly interacting NiO species. Peak 2 (~414–434 °C) corresponded to NiO moderately interacting with the SBA-16 support. Peak 3 (~478–519 °C) corresponded to NiO species interacting strongly with the support, possibly involving Ni–O–Si and/or Ni–O–Gd linkages. The relative area of this peak varied slightly between 0.5–2 wt.% Gd (24.4–27.5%) and was highest for 3 wt.% Gd (37.7%). Peak 4 (>580 °C) was associated with NiO incorporated into the silica framework or forming stable Ni–Gd–O phases. The position of Peak 4 shifted progressively to higher temperature with increasing Gd loading, while the area increased from 17.6% to about 30% at 2–3 wt.% Gd.

In the post-treatment profiles, changes were observed in both peak positions and their relative areas. For 0.5–2 wt.% Gd loadings, all peaks shifted to lower temperatures (by approximately 20–54 °C for Peak 1), and the area of Peak 1 decreased compared to the fresh state. In these samples, Peak 4 became the dominant feature (~56–60% area).

At 3 wt.% Gd, Peak 1 area increased from 6.1% to 26.9%, accompanied by a small rise in temperature, while Peak 3 area decreased from 37.7% to 10.9%. Peak 4 remained the largest contributor (~55%) but shifted to a lower temperature than in the fresh state. These results indicated that moderate Gd loadings (1–2 wt.%) maintained a balanced distribution of reducible Ni species after CO_2_ cycling, while excessive loading (3 wt.%) led to a redistribution towards both weakly and strongly bound Ni populations [32].

### 3.2. Catalytic Activity

**Figure 5:**
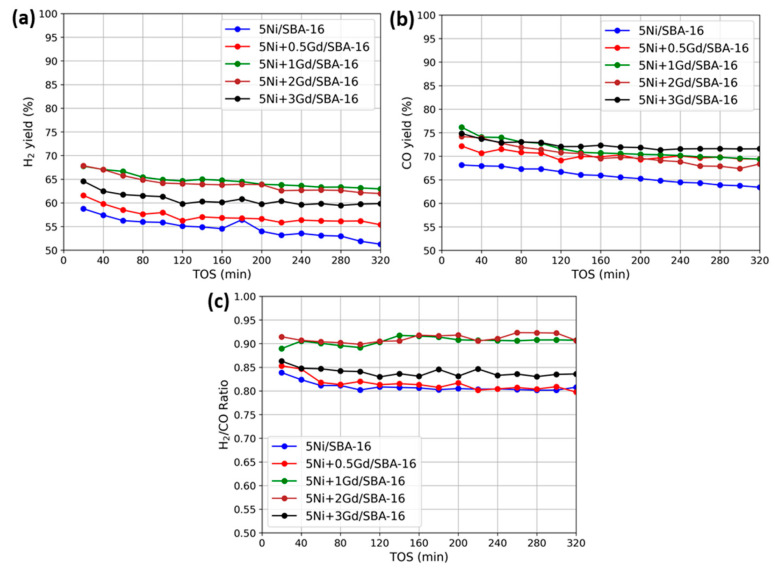
Catalytic activity results versus TOS at 800 °C (**a**) yield of H_2,_ (**b**) yield of CO, and (**c**) H_2_/CO ratio for reduced 5Ni+xGd/SBA-16 (x= 0, 0.5, 1, 2, 3 wt.%).

The catalytic performance of 5Ni/SBA-16 catalysts with varying Gd loadings was evaluated for DRM conditions at 800 °C and a GHSV of 42,000 mL g^−1^ h^−1^. The long-term performance over 320 min is depicted in Figure 5, which presents (a) the yield of H_2_, (b) the yield of CO, and (c) the molar ratio of H_2_/CO for all catalysts.

The unpromoted 5Ni/SBA-16 catalyst exhibited the lowest H_2_ yield, decreasing from 57% at the start of reaction to 53% after 320 min on-stream (TOS). The incorporation of 0.5 wt.% Gd led to an increase in the initial H_2_ yield to 62%, with a slight decline over time. The highest H_2_ yields were obtained with 1 wt.% Gd and 2 wt.% Gd catalysts, both starting at nearly 66–67% and showing minimal decrease during TOS. At 3 wt.% Gd loading, the H_2_ yield (65% initially) was lower than that of the 1–2 wt.% samples and showed a faster decline, although still higher than that of the unpromoted catalyst. The CO yield trends (Figure 5b) were similar to the H_2_ yield. The unpromoted catalyst initially showed a 68% CO yield, which decreased to 62% over TOS. The 0.5 wt.% Gd catalyst improved CO yield to 72%. The 1 wt.% and 2 wt.% Gd catalysts maintained the highest CO yields (76–73% and 75–73%, respectively). At 3 wt.% Gd, CO yield (74% initially) did not improve further and declined slightly faster than the optimum Gd-loaded samples. Overall, at the end of 320 min time on stream; H_2_-yield and CO-yield remained highest for 1–2 wt.% Gd promoted catalysts. These catalysts (5Ni+1Gd/SBA-16 and 5Ni+2Gd/SBA-16) also showed optimum CH_4_ conversion (69%) and optimum CO_2_ conversion (72–73%) at the end of 320 min time on stream (Table S1).

The molar ratio of H_2_/CO (Figure 5c) for the unpromoted catalyst started at ~0.84 and gradually declined. The 0.5 wt.% Gd catalyst had the lowest ratio (0.85) throughout the test. The 1 wt.% and 2 wt.% Gd catalysts showed the highest ratios (0.88–0.92), while the 3 wt.% Gd catalyst was intermediate (0.82–0.86).

Table S2 (Supplementary Information) summarizes the catalytic performance of the 5Ni+xGd/SBA-16 catalysts (x = 1 and 2 wt.%) along with various other promoters and Ni/SBA-16 systems reported in the literature. At 800 °C and a GHSV of 42,000 mL·g^−1^·h^−1^, the 1–2 wt.% Gd–Ni/SBA-16 catalyst gave comparable H_2_ and CO yield with a H_2_/CO ratio of 0.92. These results are comparable to those previously reported for Ni/SBA-16 systems.

**Figure 6:**
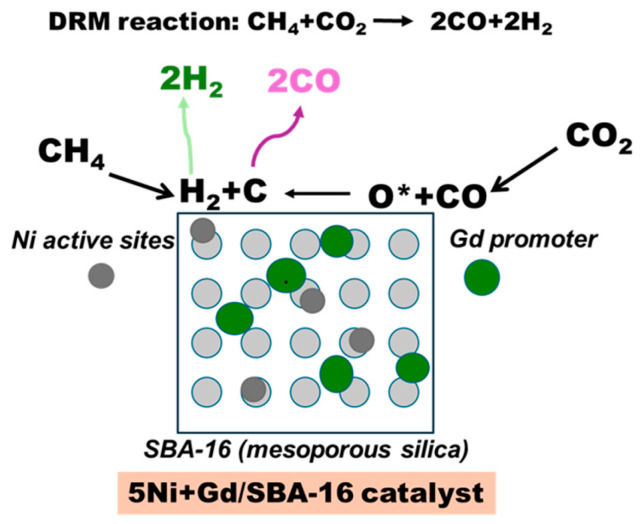
Proposed reaction mechanism for DRM over Gd-promoted Ni/SBA-16 catalysts. The Ni active sites (grey circles) facilitate CH_4_ activation, while Gd promoter species (green circles) enhance CO_2_ adsorption and oxygen mobility, forming intermediates (H_2_ + C) and (O* + CO) that yield syngas (2H_2_ + 2CO). The asterisk (O *) represents active surface oxygen species participating in the DRM reaction.

The better performance of the 1 wt.% and 2 wt.% Gd catalysts can be correlated with earlier characterization results. XPS showed a higher fraction of Si–O–M linkages at 1–2 wt.%, indicating stronger metal–support interactions. Initial and post-treatment H_2_–TPR analyses showed that these catalysts retained a balanced distribution of reducible Ni species after treatment, which is favourable for stable activity. In contrast, 3 wt.% Gd showed reduced Si–O–M linkages and an increased fraction of weakly interacting NiO and reduced strongly bound species. Figure 6 presents the proposed reaction mechanism for DRM over Gd-promoted Ni/SBA-16 catalysts, highlighting the roles of Ni active sites and Gd promoter over the mesoporous SBA-16 support.

### 3.3. Design of Experiments and Model Validation

A suitable power transformation (Equation (5)) was incorporated within the CCD framework to stabilize variance and improve model fit [33]. ANOVA results (Table S3—Supporting Information) indicated that all model terms were statistically significant (*p* < 0.05), while high coefficients of determination (R^2^ = 99.54% for H_2_ yield and 99.41% for CO yield) and confirmed strong agreement between experimental and model-predicted values. The correlation in predicted vs. observed plots (Figure S2) further confirmed model reliability [22,34,35]. By applying ANOVA at a significance level of α = 0.05 and using the Stat-Ease package, the experimental data were analysed, and the following models were proposed.

(6) $$H_{2}Yield(\%)\;=-\;804.87256+1.97534\;T\;-\;0.002679\;V+154.79755\;R+0.00000351104\;T\;V\;-\;0.119507\;T\;R\;-\;0.000361\;V\;R\;-\;0.001097\;T^{2}\;-\;0.00000000539685\;V^{2}\;-\;39.31307\;R^{2}$$

(7) $$CO\;Yield\left(\%\right)=-942.0243+2.46091\;T\;-\;0.002235\;V+72.10662\;R+0.00000358111\;T\;V\;\;-0.089684\;T\;R-0.000265\;V\;R-\;0.001461\;{\;T}^{2}-0.00000000109012\;V^{2}-\;4.91681\;R^{2}\;$$

The models (6) and (7) consist of multiple components: the intercept terms (β_0_), which indicate the expected value of the response when all independent variables are set to zero, while the main effects ($\sum_{i=1}^{3}\beta_{i}X_{i}$), indicating the linear impact of each factor on the response while holding the others constant; the interaction terms ($\sum_{i=1}^{2}\sum_{j=i+1}^{3}\beta_{ii}X_{i}X_{j}$), which represent the combined influence of two interacting variables; and the quadratic terms ($\sum_{i=1}^{3}\beta_{ii}X_{i}^{2}$), which account for curvature or nonlinear effects in the response.

#### Response Surface Analysis and Optimization of Process Parameters

**Figure 7:**
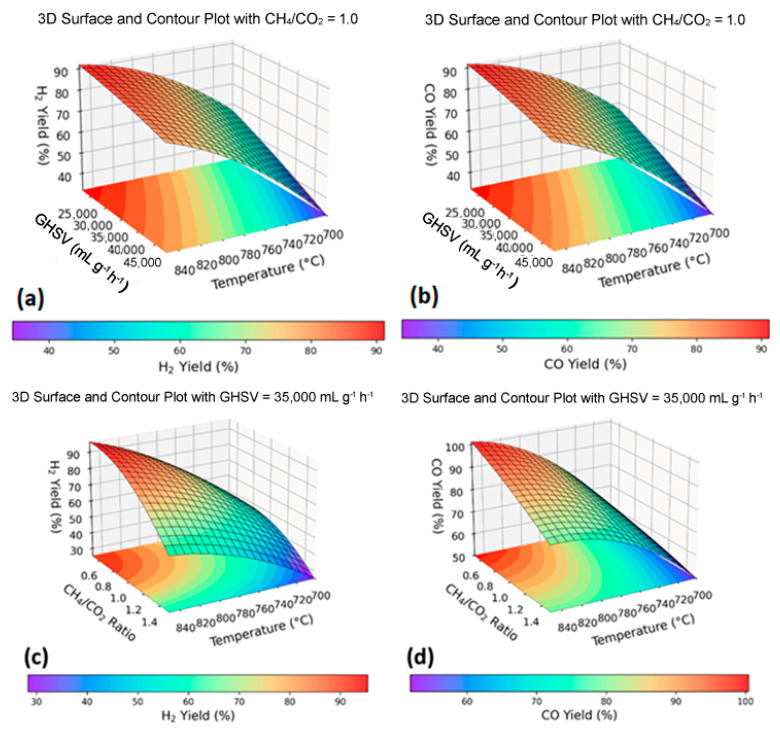
RSM-3D surface and contour plots depicting the interactive effects of process variables on H_2_ and CO yields during DRM (**a**) H_2_ yield as a function of temperature and GHSV at a fixed CH_4_/CO_2_ ratio of 1, (**b**) CO yield as a function of temperature and GHSV at a fixed CH_4_/CO_2_ ratio of 1, (**c**) H_2_ yield as a function of temperature and CH_4_/CO_2_ ratio at a fixed GHSV of 35,000 mL g^−1^ h^−1^, and (**d**) CO yield as a function of temperature and CH_4_/CO_2_ ratio at a fixed GHSV of 35,000 mL g^−1^ h^−1^.

Three-dimensional (3D) response surface plots derived from the models are presented in Figure 7 to visualize the interactive effects of GHSV, temperature, and CH_4_/CO_2_ ratio on H_2_ and CO yields during DRM. These visualizations highlight the interactive effects of process variables, enabling identification of optimal operating conditions.

When the CH_4_/CO_2_ ratio was fixed at 1 (Figure 7a), H_2_ yield improved with rising temperature and with decrease in GHSV. Under these conditions, H_2_ yield enhanced from 42.1% at 700 °C to 95.09% at 850 °C for a GHSV—32,000 mL g^−1^ h^−1^. Similarly, at GHSV fixed at 35,000 mL g^−1^ h^−1^ (Figure 7c), increasing the temperature and lowering the CH_4_/CO_2_ ratio further enhanced H_2_ yield.

For the CO yield, a similar trend was observed. At a CH_4_/CO_2_ ratio of 1 (Figure 7b), CO yield increased from 42.12% at 700 °C to 95.09% at 850 °C at a GHSV of 32,000 mL g^−1^ h^−1^. Similarly, at a constant GHSV of 35,000 mL g^−1^ h^−1^ (Figure 7d), higher temperatures and lower CH_4_/CO_2_ ratios promoted higher CO yields. These results indicate that elevated temperatures and reduced GHSV favour endothermic DRM kinetics and improve reactant–surface interactions. Additional 2D contour plots (Figure S3, Supplementary Information) are provided for complementary visualization of these trends.

Numerical optimization using the Stat-Ease software identified the optimal process parameters for maximizing H_2_ yield. The solution with the highest desirability index yielded the following theoretical conditions: temperature = 848.9 °C, GHSV = 31, 283 mL g^−1^ h^−1^, and CH_4_/CO_2_ ratio = 0.61, predicting a H_2_ yield of 96.64%. Experimental validation in these conditions produced a 96.66% yield, confirming the accuracy of the model. The theoretical and experimental optimum conditions are summarized in Table 5.

The optimized process parameters identified through RSM modelling offered complementary insight into the potential performance of the catalysts under varied operating conditions. The catalytic activity section focused on fixed reaction parameters (temperature 800 °C and GHSV = 42,000 mL g^−1^ h^−1^). This model extended this understanding by predicting conditions that could further enhance H_2_ and CO yields.

### 3.4. TGA–DSC and Raman Characterization of Coke Deposition on Spent Catalyst

The TGA and Raman analyses of the spent 5Ni+2Gd/SBA-16 under optimized reaction conditions (presented in Table 5) were carried out in order to investigate the presence of carbon deposition on the catalyst surface. The TGA–DSC profile of the spent 5Ni+2Gd/SBA-16 catalyst (Figure S4, Supplementary Information) exhibited a gradual weight loss of ~1.76% in the temperature range 580–780 °C. This can be attributed to the oxidation of carbonaceous deposits (coke) formed during the catalytic reaction. The corresponding DSC curve, however, did not exhibit a distinct exothermic peak; instead, it displayed a steady downward drift. This behaviour suggests that the heat released during coke combustion is small in magnitude and distributed over a broad temperature range. The absence of a well-defined DSC peak indicates the presence of a relatively small amount of surface coke undergoing slow oxidation [36].

The Raman spectrum of the spent catalyst (Figure S5, Supplementary Information) showed characteristic peaks associated with carbonaceous deposits. The D band (~1350 cm^−1^) corresponds to disordered or defect sites in carbon, while the G band (~1580 cm^−1^) arises from the in-plane stretching vibrations of sp^2^–sp^2^-hybridized carbon domains, indicating the presence of graphitic structures. Additionally, the 2D band (~2700 cm^−1^), a second-order overtone of the D band, reflected the graphitic ordering and stacking of sp^2^ carbon layers [37]. The relative intensities of these peaks suggested that the deposited carbon is a mixture of amorphous and graphitic forms [38].

The combined TGA–DSC and Raman results indicated that the spent catalyst 5Ni+2Gd/SBA-16 under optimized conditions accumulated a limited amount of coke, majorly graphitic in nature, which oxidizes slowly over a wide temperature range.

## 4. Conclusions

Gd-promoted Ni/SBA-16 catalysts (0.5–3 wt.%) were evaluated for DRM at 800 °C with a GHSV of 42,000 mLg^−1^ h^−1^. The 5Ni/SBA-16 catalysts with 1–2 wt.% Gd loading exhibited the best performance, achieving stable H_2_ yields of ~67% and CO yields of 73–76%, with H_2_/CO ratios of 0.88–0.91 sustained over 320 min of time-on-stream. XPS, together with initial and post-treatment H_2_–TPR, indicated stronger Ni–support interactions and a favourable distribution of reducible Ni species at these loadings. CCD-RSM gave well-fitted models (R^2^ = 0.9954 for H_2_, 0.9941 for CO) and identified optimum conditions of 848.9 °C, 31,283 mL g^−1^ h^−1^, and CH_4_/CO_2_ = 0.61, where the predicted H_2_ yield (96.64%) was very close to the experimental value (96.66%). Combined TGA–DSC and Raman analyses of the spent 5Ni+2Gd/SBA-16 catalyst under optimized conditions exhibited limited coke deposition, mainly graphitic in nature. These findings highlight the synergistic role of rare-earth promotion and process parameter optimization in enhancing DRM efficiency and providing a mechanistic basis for future catalyst design.

## Figures and Tables

**Table 1:** Process parameters for the CCD modeling approach, showing both the actual experimental ranges (temperature, GHSV, and CH_4_/CO_2_ ratio) and their corresponding coded values.

Process Parameters	Levels	Levels
	−1 (Low)	+1(High)
GHSV: Gas hourly space velocity (mL g^−1^ h^−1^)	22,000	48,000
T: Temperature (°C)	700	850
R: CH_4_/CO_2_	0.5	1.5

**Table 2:** Central Composite Design (CCD) matrix for three factors (temperature, GHSV, CH_4_/CO_2_). The table shows coded values and corresponding experimental runs.

Run	T (Coded)	SV (Coded)	R (Coded)	Temperature (°C)	GHSV (mL g^−1^ h^−1^)	CH_4_/CO_2_
1	+1.000	−1.000	+1.000	850	22,000	1.25
2	+1.000	−1.000	−1.000	850	22,000	0.75
3	−1.000	−1.000	+1.000	700	22,000	1.25
4	−1.000	−1.000	−1.000	700	22,000	0.75
5	0.000	−1.000	0.000	775	22,000	1.00
6	+1.000	0.000	0.000	850	35,000	1.00
7	0.000	0.000	+1.000	775	35,000	1.25
8	−1.000	0.000	0.000	700	35,000	1.00
9	0.000	0.000	0.000	775	35,000	1.00
10	0.000	0.000	−1.000	775	35,000	0.75
11	0.000	+1.000	0.000	775	48,000	1.00
12	+1.000	+1.000	+1.000	850	48,000	1.25
13	+1.000	+1.000	−1.000	850	48,000	0.75
14	−1.000	+1.000	+1.000	700	48,000	1.25
15	−1.000	+1.000	−1.000	700	48,000	0.75
16	+0.333	+1.000	−1.000	800	48,000	0.75
17	+0.333	0.000	0.000	800	35,000	1.00

**Table 3:** (**a**). Si 2p XPS binding energies and relative peak areas for 5Ni+xGd/SBA-16 (x = 0, 0.5, 1, 2, 3 wt.%) catalysts. (**b**). O 1s XPS binding energies and relative peak areas corresponding to 5Ni+xGd/SBA-16 (x = 0, 0.5, 1, 2, 3 wt.%) catalysts.

(**a**)						
**Peak** **Assignment**	**Parameter**	**5Ni/SBA-16**	**5Ni+0.5Gd/SBA-16**	**5Ni+1Gd/SBA-16**	**5Ni+2Gd/SBA-16**	**5Ni+3Gd/SBA-16**
Si–O–M	B.E. (eV)	104.1	104.4	104.7	105.0	Not observed
	% area	8.1	33.4	18.7	19.6	Not observed
Si–O–Si	B.E. (eV)	103.3	103.6	103.8	103.7	103.8
	% area	16.1	19.7	28.5	24.1	37.9
Si–O–H	B.E. (eV)	102.4	102.6	102.9	102.6	102.5
	% area	42.1	26.1	37.0	30.5	16.8
SiOx (x < 2)	B.E. (eV)	100.8	100.4	100.7	101.7	101.3
	% area	33.7	20.9	15.8	25.8	19.4
(**b**)						
Peak Assignment	Parameter	5Ni/ SBA-16	5Ni+0.5Gd/SBA-16	5Ni+1Gd/SBA-16	5Ni+2Gd/SBA-16	5Ni+3Gd/SBA-16
Si–O–Si	B.E. (eV)	533.8	533.8	533.4	533.4	532.9
	% area	21.8	32.3	26.1	28.5	25.3
Si–O–M	B.E. (eV)	532.9	532.7	532.4	532.1	531.9
	% area	17.6	23.2	19.4	31.3	16.9
C–O / C=O	B.E. (eV)	532.0	531.7	531.4	531.4	531
	% area	33.8	19.7	35.8	22.6	7.6
M–O–M	B.E. (eV)	530.1	530.1	529.0	530.6	529.6
	% area	26.8	24.9	18.6	17.6	50.1

**Table 4:** Peak temperatures and corresponding areas for H_2_–TPR profiles of fresh and post–CO_2_-TPD 5Ni + xGd/SBA-16 catalysts (x= 0, 0.5, 1, 2, 3 wt.%).

Catalyst	Peak	Temp. (°C) Fresh	% Area Fresh	Temp. (°C) After CO_2_-TPD	% Area After CO_2_-TPD
5Ni+0.5Gd/SBA-16	1	390.4	32.3	355.0	10
	2	435.2	25.8	394.9	12.7
	3	486.9	24.4	446.0	17.8
	4	580.9	17.6	528.1	59.5
5Ni+1Gd/SBA-16	1	386.5	25.7	332.3	11.5
	2	431.4	23.7	395.2	10.2
	3	481.3	27.5	451.9	20.1
	4	589.6	23.1	544.4	58.2
5Ni+2Gd/SBA-16	1	377.7	7.7	343.6	6.8
	2	414.0	36.4	390.0	6.4
	3	478.6	25.1	456.8	31.1
	4	598.0	30.8	565.3	55.7
5Ni+3Gd/SBA-16	1	393.1	6.1	401.9	26.9
	2	434.1	25.9	453.4	7.1
	3	509.9	37.7	492.8	10.9
	4	638.0	30.3	571.6	55.1

**Table 5:** Theoretical optimum and experimental conditions for process optimization, showing the key variables (temperature, GHSV, and CH_4_/CO_2_ ratio), their corresponding goals, and the resulting H_2_ yield.

Variables	Goal Function	Theoretical Optimum	Experimental Validation
Temperature (°C)	700–850	848.9	845
GHSV (mL g^−1^ h^−1^)	22,000–48,000	31,283	31,283
CH_4_/CO_2_ ratio	0.5–15	0.61	0.61
H_2_ yield (%)	Maximum	96.64	96.66

## Data Availability

All data that support the findings of this study are included within the article.

## Supplementary Materials

The following supporting information can be downloaded at: https://www.mdpi.com/article/10.3390/nano15191527/s1, Catalyst characterization S1; Model accuracy S2; Figure S1. FTIR spectra of 5Ni–xGd/SBA-16 catalysts (x = 0, 0.5, 1.0, 2.0, 3.0 wt.%) showing surface –OH vibrations and Si–O–Si bands; Figure S2. Comparison between the actual and estimated data for the response variables; Figure S3. Two-dimensional relationship between factors (Temperature (T), Space Velocity (GHSV), and ${CH}_{4}/{CO}_{2}$) and H_2_ yield; Figure S4. TGA–DSC of the spent 5Ni+2Gd/SBA-16 catalyst (under optimized conditions) showing 1.76% weight loss from 580–780 °C due to slow coke oxidation, with no distinct exothermic DSC peak; Figure S5. Raman spectrum of the spent catalyst showing peak-fitted carbon features: The D band (~1350 cm^−1^), the G band (~1580 cm^−1^), and the 2D band (~2700 cm^−1^); Table S1. Performance data of the studied catalysts [7,11,12,13,14,15,39,40]; Table S2. Comparison of the DRM performance of Ni/SBA-16-based catalysts from this work and the literature; Table S3. Analysis of variance (ANOVA) for the quadratic models of the three response variables.
