# Supplementary material for: Dry Reforming of Methane Using Gd-promoted Ni/SBA-16 Catalyst: Structure, Activity and Process Optimization with Response Surface Methodology
Source: Nanomaterials (Basel). 2025 Oct 6;15(19):1527. doi: 10.3390/nano15191527 (PMC12526237; doi:10.3390/nano15191527)
Supplement: Supplementary file 1 [file nanomaterials-15-01527-s001.zip › nanomaterials-3898097-supplementary.pdf]

Article

Dry Reforming of Methane Using Gd-promoted Ni/SBA-16 Catalyst: Structure, Activity and Process Optimization with Response Surface Methodology

Salma A. Al-Zahrani [1,2,*], Mohammed F. Alotibi [3,*], Ahmed I. Osman [4], Ahmed A. Bhran [5], Maha Awjan Alreshidi [1,2], Ahmed Al Otaibi [1,2], Hessah Difallah A. Al-Enazy [1,2], Nuha Othman S Alsaif [1,2] and Ahmed S. Al-Fatesh [6,*]

[1] Chemistry Department, Faculty of Science, University of Ha'il, P.O. Box 2440, Ha'il 81451, Saudi Arabia; m.alrashedi@uoh.edu.sa (M.A.A.); ahmed.alotaibi@uoh.edu.sa (A.A.O.); h.alenazy@uoh.edu.sa (H.D.A.A.-E.); n.alseif@uoh.edu.sa (N.O.S.A.)
[2] Scientific and Engineering Research Center, University of Ha'il, Ha'il 2440, Saudi Arabia
[3] Institute of Refining and Petrochemicals Technologies, King Abdulaziz City for Science and Technology (KACST), P.O. Box 6086, Riyadh 11442, Saudi Arabia
[4] School of Engineering, Technology, and Design, Canterbury Christ Church University, Canterbury CT1 1QU, UK; ahmed.osman@canterbury.ac.uk
[5] Chemical Engineering Department, College of Engineering, Imam Mohammad Ibn Saud Islamic University (IMSIU), Riyadh 11432, Saudi Arabia; aabahran@imamu.edu.sa
[6] Chemical Engineering Department, College of Engineering, King Saud University, P.O. Box 800, Riyadh 11421, Saudi Arabia
* Correspondence: s.alzahrane@uoh.edu.sa (S.A.A.-Z.); mfalotaibi@kacst.gov.sa (M.F.A.); alfatesh@ksu.edu.sa (A.S.A.-F.)

Catalyst characterization S1

X-ray diffraction (XRD) analysis was performed on a Bruker D8 Advance diffractometer (Bruker, Apex II) equipped with Cu Kα radiation and a nickel filter, operating at 40 kV and 40 mA. Data were collected over a 2θ range of 5–100° with a step size of 0.01°.

The Micromeritics Tristar II 3020 instrument (N_2 physisorption at − 196 °C, degassing for 4 h at 200 °C) was used to determine the N_2 adsorption-desorption profile to obtain the specific surface area, total pore volume and average pore diameter of the synthesized catalysts. The Brunauer-Emmet-Teller (BET) equation estimated the surface area, while the non-local density function model estimated the pore size distribution. Fourier-transform infrared spectroscopy (FTIR, Shimadzu IRPrestige-21, Japan) was used to identify functional groups on the catalyst surface, with spectra collected in the range of 400–4000 cm^{-1} using KBr pellets. Chemical surface analysis was carried out using X-ray photoelectron spectroscopy (XPS, Thermo Fisher Scientific, USA) with Al Kα radiation. Scans were performed over the ranges of 395–415 eV, 526–540 eV, and 840–900 eV, with energy steps of 5, 2, and 10 eV, respectively, using a pass energy of 200 eV.

Temperature-programmed reduction (H_2-TPR) and CO_2 temperature-programmed desorption (CO_2-TPD) experiments were conducted using a Micromeritics AutoChem II 2920 system fitted with a thermal conductivity detector (TCD). For H_2-TPR, ~70 mg of sample was exposed to 10% H_2/He (40 mL min^{-1}) and

heated from room temperature to 900 °C at a rate of 10 °C min^{-1}. A cold trap was used to remove water generated during reduction, and hydrogen uptake was monitored with the TCD.

After the reduction step, CO_2-TPD was carried out on the same specimen. The catalyst was saturated with 10% CO_2/He (30 mL min^{-1}) at 50 °C for 30 minutes, followed by heating to 800 °C at 10 °C min^{-1} while recording CO_2 desorption via the TCD. Subsequently, a second H_2-TPR run was performed under identical operating conditions to complete the redox cycle.

The amount of carbon deposition on best performing spent catalysts was quantified by thermogravimetric analysis (TGA) using a Shimadzu TGA-51. Approximately 10–15 mg of used catalyst in a platinum pan was heated from room temperature to 1000 °C at 20 °C min^{-1} under air flow while continuously monitoring the mass change.

Raman spectroscopy was performed on the best performing catalyst with a Raman spectrometer (JASCO, Japan) equipped with Spectra Manager v2 software. Excitation employed a 532 nm laser, scanning the 150–3000 cm^{-1} range. The laser intensity was set to 1.6 mW, with 10 s exposure time and three accumulations for each spectrum.

Model accuracy S2

The accuracy of the predicted values from the fitted models, developed using the Central Composite Design (CCD) methodology, was assessed using mathematical metrics such as R^2, where a higher R^2 value indicates a better model fit, and the model more accurately describes the relationship between the variables. Absolute percentage error (APE), mean absolute percentage error (MAPE), and mean absolute error (MAE) are used also to test the model accuracy. These statistical amounts as presented in **Eqs. S1 to S5**. Furthermore, a comparison between the actual and predicted data was plotted, demonstrating a strong agreement, which indicates the reliability of the models.

$$R^2 = \frac{SS_{model}}{SS_{Total}} = 1 - \frac{SS_{Error}}{SS_{Total}} \quad (S1)$$

$$\underbrace{\sum_{i=1}^{n}(E_i - \bar{E})^2}_{SS_{Total}} = \underbrace{\sum_{i=1}^{n}(P_i - \bar{E})^2}_{SS_{model}} + \underbrace{\sum_{i=1}^{n}(E_i - P_i)^2}_{SS_{Error}} \quad (S2)$$

where E_i and P_i are the experimental/actual and predicted values of the i^{-th} observation, $\bar{E}$ is the mean value of the response variable across all observations, and n is the number of experiments.

$$APE = 100 * \frac{|E_i - P_i|}{E_i} \% \quad (S3)$$

$$MAE = \frac{1}{n}\sum_{i=1}^{n}\frac{|E_i - P_i|}{E_i} \quad (S4)$$

$$MAPE = 100 * \frac{1}{n}\sum_{i=1}^{n}\frac{|E_i - P_i|}{E_i} \% \quad (S5)$$

Figure S1 illustrates agreement, between the actual and predicted response variable values which align closely with the fitted line $X = Y$. For a good fit, data points should be near this line, with narrow confidence bands. Notably, points far from the mean have a greater influence on the fitted line, while those far from the line itself may indicate outliers, both of which can negatively impact the model's accuracy.

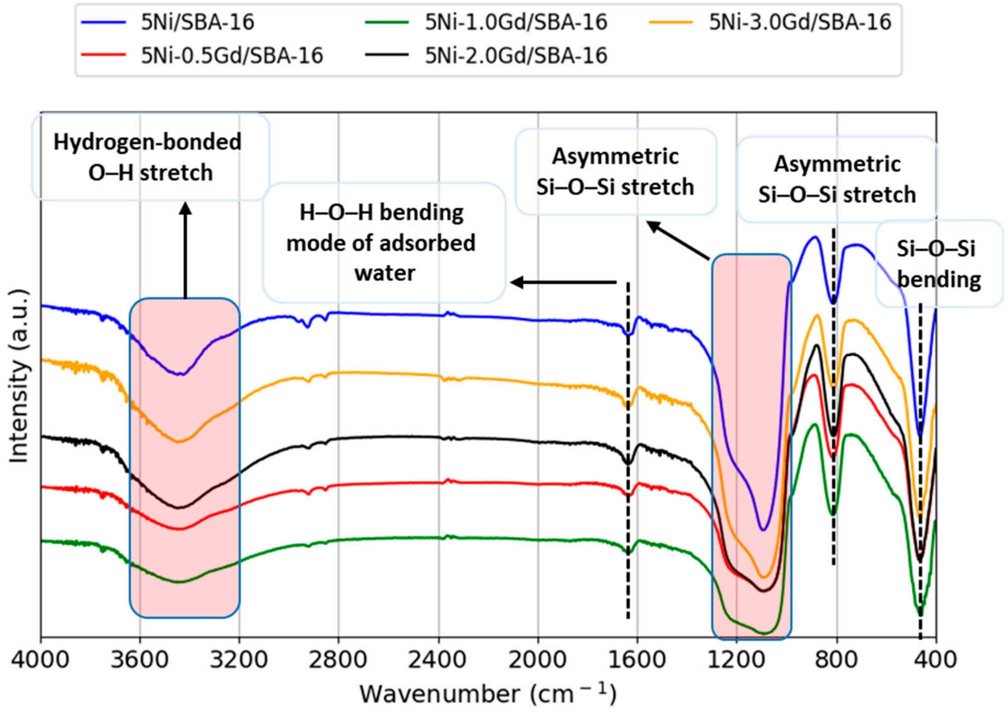

Figure S1. FTIR spectra of 5Ni–xGd/SBA-16 catalysts (x = 0, 0.5, 1.0, 2.0, 3.0 wt. %) showing surface –OH vibrations and Si–O–Si bands

Table S1. Performance data of the studied catalysts

Catalyst	CH_4 Conversion (%)		CO_2 Conversion (%)	
	Initial	Final*	Initial	Final*
**5Ni/SBA-16**	62.6	57.6	73.7	68.9
**5Ni+0.5Gd/SBA-16**	63.2	56.9	74.5	71.0
**5Ni+1Gd/SBA-16**	69.2	63.2	78.6	73.4
**5Ni+2Gd/SBA-16**	68.9	62.7	76.9	71.9
**5Ni+3Gd/SBA-16**	66.3	60.3	75.9	72.7

*After reaction span of 320 min

Table S2. Comparison of the DRM performance of Ni/SBA-16-based catalysts from this work and the literature

	Catalyst system	T (°C)	GHSV (mL g^{-1} h^{-1})	CH_4 conv. (%)	CO_2 conv. (%)	H_2 yield (%)	CO yield (%)	H_2/CO ratio	References
1	Gd–Ni/SBA-16	800	42,000	—	—	~67	~76	0.92	Present work
2	5Ni1Gd/LaZr	700	42,000	—	—	80	—	—	[1]
3	5Ni1Ga/LaZr	700	42,000	—	—	73	—	—	[1]
4	5Ni/LaZr	700	42,000	—	—	58	—	—	[1]
5	5Ni4Gd/Y + Zr	800	42,000	—	—	80	—	—	[2]
6	5Ni4Ho/YZr	700	42,000	—	—	84	—	—	[3]
7	5Ni2.5Ce/LaZr	700	42,000	—	—	87	—	—	[4]
8	Ni/SBA-16-WI	700	24,000	62.8	73.1	—	—	0.82	[5]
9	Ni/SBA-16-CA	700	24,000	48.0	57.1	—	—	0.76	[5]
10	Ni/SBA-16-AE	700	24,000	82.3	87.3	—	—	0.88	[5]
11	Ni/SBA-16	700	45,000	73	~75	—	—	~0.7	[6]
12	Ce-Ni/SBA-16	700	45,000	72	~74	—	—	~0.7	[6]
13	Ni/SBA-16–MgO	700	12,000	55	~65	—	—	~0.7	[7]
14	Ce–Ni/SBA-16–MgO	700	12,000	65	~70	—	—	~0.7	[7]
15	Y–Ni/SBA-16–MgO	700	12,000	74	~80	—	—	~0.8	[7]
16	La–Ni/SBA-16–MgO	700	12,000	31	~40	—	—	~0.5	[7]
17	Ni/SBA-16	800	36,000	~65	~72	—	—	~0.75	[8]
18	Ni/SBA-16–N (N-modified)	800	36,000	~72	~78	—	—	~0.80	[8]

**References*

[1] Al-Fatesh, A. S.; Khatri, J.; Kumar, R.; Kumar Srivastava, V.; Osman, A. I.; AlGarni, T. S.; Ibrahim, A. A.; Abasaeed, A. E.; Fakeeha, A. H.; Rooney, D. W. Role of Ca, Cr, Ga and Gd Promotor over Lanthana-Zirconia–Supported Ni Catalyst towards H_2-Rich Syngas Production through Dry Reforming of Methane. *Energy Science & Engineering* **2022**, *10*, 866–880. DOI: 10.1002/ese3.1063.

[2] Fakeeha, A. H.; Al-Fatesh, A. S.; Srivastava, V. K.; Ibrahim, A. A.; Abahussain, A. A. M.; Abu-Dahrieh, J. K.; Alotibi, M. F.; Rawesh, R. Hydrogen production from gadolinium-promoted yttrium–zirconium-supported Ni catalysts through dry methane reforming. *ACS Omega* **2023**, *8*, 22108. DOI: 10.1021/acsomega.3c02229.

[3] Fakeeha, A. H.; Patel, R.; El Hassan, N.; Al-Zahrani, S. A.; AlAwadi, A. S.; Frusteri, L.; Bayahia, H.; Alharth, A. I.; Al-Fatesh, A. S.; Kumar, R. Holmium Promoted Yttria-Zirconia Supported Ni Catalyst for H_2 Production via Dry Reforming of Methane. *International Journal of Hydrogen Energy* **2022**, *47*, 38242–38257. DOI: 10.1016/j.ijhydene.2022.09.029.

[4] Khatri, J.; Al-Fatesh, A. S.; Fakeeha, A. H.; Ibrahim, A. A.; Abasaeed, A. E.; Kasim, S. O.; Osman, A. I.; Patel, R.; Kumar, R. Ce Promoted Lanthana-Zirconia Supported Ni Catalyst System: A Ternary Redox System for Hydrogen Production. *Molecular Catalysis* **2021**, *504*, 111498. DOI: 10.1016/j.mcat.2021.111498.

[5] Sun, C.; Wang, Z.; Li, J.; Chen, J.; Zhang, Q.; He, H.; Zhang, H.; Luo, J. Boosting CO_2 reforming of methane via the metal–support interaction in mesostructured SBA-16-derived Ni nanoparticles. *Applied Materials Today* **2022**, *26*, 101354. DOI: 10.1016/j.apmt.2021.101354.

[6] Zhang, S.; Muratsugu, S.; Ishiguro, N.; Tada, M. Ceria-doped Ni/SBA-16 catalysts for dry reforming of methane. *ACS Catalysis* **2013**, *3*, 1855–1864. DOI: 10.1021/cs400159w.

[7] Taherian, Z.; Khataee, A.; Orooji, Y. Nickel-based nanocatalysts promoted over MgO-modified SBA-16 for dry reforming of methane for syngas production: Impact of support and promoters. *Journal of Energy Institute* **2021**, *97*, 100–108. DOI: 10.1016/j.joei.2021.04.005.
[8] Huo, M.; Li, L.; Zhao, X.; Zhang, Y.; Li, J. Synthesis of Ni-based catalysts supported on nitrogen-incorporated SBA-16 and their catalytic performance in the reforming of methane with carbon dioxide. *Journal of Fuel Chemistry and Technology* **2017**, *45*, 172–181. DOI: 10.1016/S1872-5813(17)30012-9.

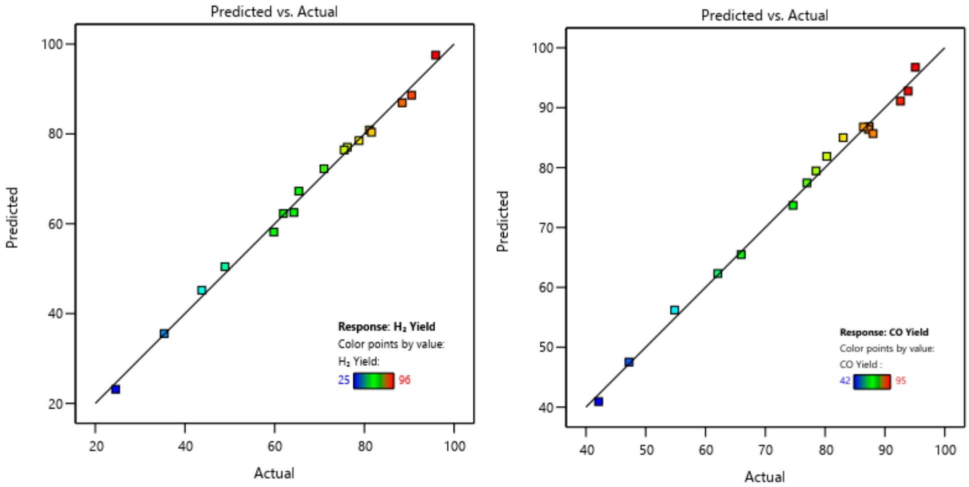

Figure S2. Comparison between the actual and estimated data for the response variables

Simulation using Design-Expert Software -2D Presentation

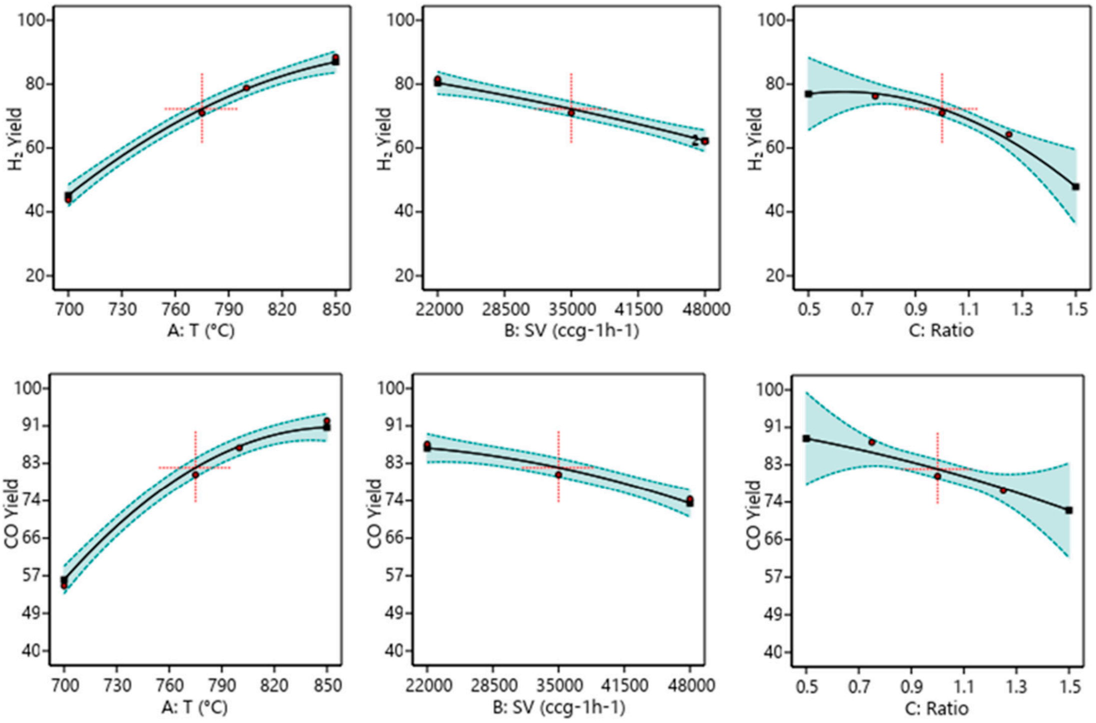

Figure S3. 2D relationship between factors (Temperature (T), Space Velocity (GHSV), and CH_4/CO_2) and H_2 yield

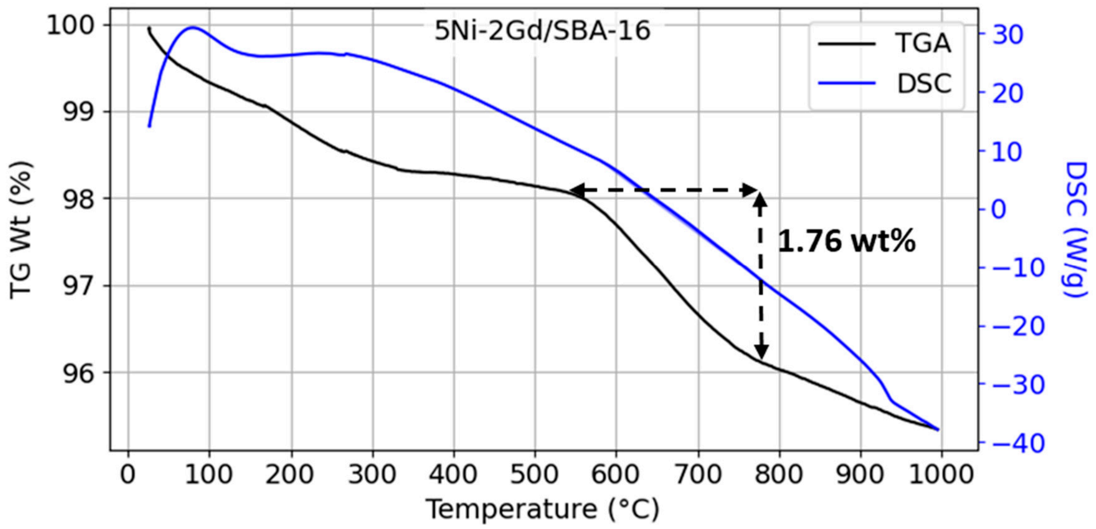

Figure S4- TGA–DSC of the spent 5Ni+2Gd/SBA-16 catalyst (under optimized conditions) showing 1.76 % weight loss from 580–780 °C due to slow coke oxidation, with no distinct exothermic DSC peak

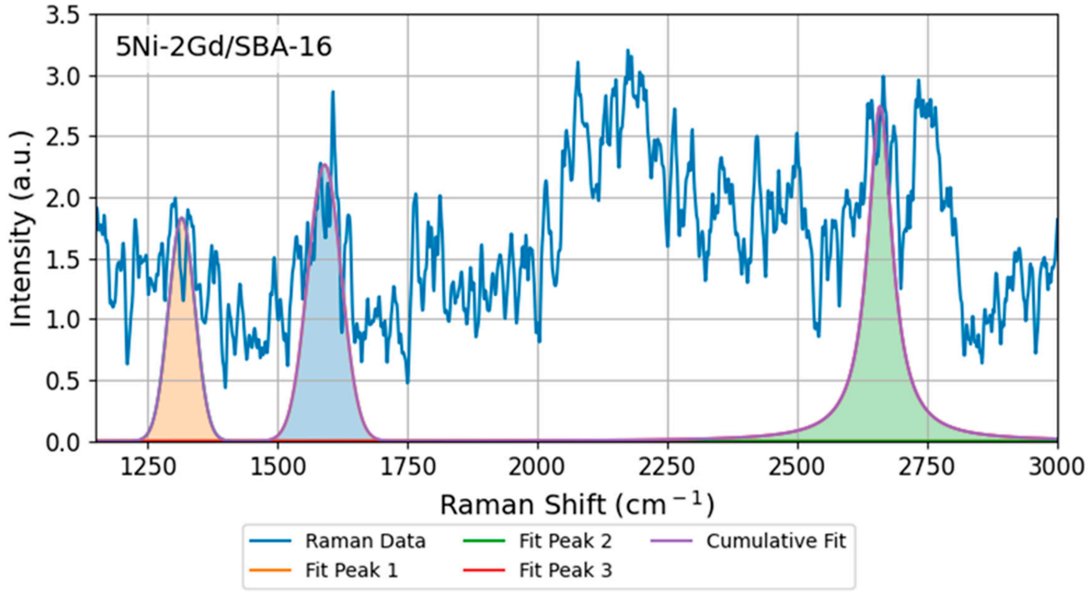

Figure S5. Raman spectrum of the spent catalyst showing peak-fitted carbon features: the D band (~1350 cm^{-1}), the G band (~1580 cm^{-1}), and the 2D band (~2700 cm^{-1})

Table S3: Analysis of variance (ANOVA) for the quadratic models of the three response variables

Response 1: H_2 Yield model with (R^2= 0.9954)					
**Source**	**Sum of Squares**	**df**	**Mean Square**	**F-value**	**p-value**
**Model**	6299.82	9	699.98	168.06	< 0.0001
A-T	4423.7	1	4423.7	1062.09	< 0.0001
B-SV	869.87	1	869.87	208.85	< 0.0001
C- CH_4/CO_2	561.36	1	561.36	134.78	< 0.0001
AB	94.52	1	94.52	22.69	0.0021
AC	40.5	1	40.5	9.72	0.0169
**BC**	11.87	1	11.87	2.85	0.1352
A^2	113.26	1	113.26	27.19	0.0012
B^2	2.3	1	2.3	0.5512	0.482
C^2	16.66	1	16.66	4	0.0856

Response 2: CO Yield model with (R^2= 0.9941)					
**Source**	**Sum of Squares**	**df**	**Mean Square**	**F-value**	**p-value**
**Model**	4238.45	9	470.94	131.24	< 0.0001
A-T	3089.38	1	3089.38	860.95	< 0.0001
B-SV	426.93	1	426.93	118.98	< 0.0001
C- CH_4/CO_2	180.64	1	180.64	50.34	0.0002
AB	98.34	1	98.34	27.4	0.0012
AC	22.81	1	22.81	6.36	0.0397
**BC**	6.38	1	6.38	1.78	0.224
A^2	200.79	1	200.79	55.95	0.0001
B^2	9.37	1	9.37	2.61	0.1502
C^2	0.2606	1	0.2606	0.0726	0.7953

Where T: temperature, GHSV: Gas Hourly Space velocity and C= $\frac{CH_4}{CO_2}$ ratio.
